# Shine a light: Under-ice light and its ecological implications in a changing Arctic Ocean

**DOI:** 10.1007/s13280-021-01662-3

**Published:** 2021-11-25

**Authors:** Giulia Castellani, Gaëlle Veyssière, Michael Karcher, Julienne Stroeve, S. Neil Banas, A. Heather Bouman, S. Andrew Brierley, Stacey Connan, Finlo Cottier, Fabian Große, Laura Hobbs, Christian Katlein, Bonnie Light, David McKee, Andrew Orkney, Roland Proud, Vibe Schourup-Kristensen

**Affiliations:** 1 Alfred-Wegener-Institute Helmholtz-Zentrum für Polar und Meeresforschung, Am Handelshafen 12, 27570 Bremerhaven, Germany; 2 British Antarctic Survey, High Cross Madingley Road, Cambridge, CB3 0ET UK; 3 Ocean Atmosphere Systems GmbH, Tewessteg 4, 20249 Hamburg, Germany; 4 University College London, Gower St, London, WC1E 6BT UK; 5 University of Manitoba, 66 Chancellors Cir, Winnipeg, MB R3T 2N2 Canada; 6 National Snow and Ice Data Center CIRES, 449 UCB University of Colorado, Boulder, CO 80309-0449 USA; 7 University of Strathclyde, Livingstone Tower, 26 Richmond Street, Glasgow, G1 1XH UK; 8 University of Oxford, South Parks Road, Oxford, OX1 3AN UK; 9 Pelagic Ecology Research Group, Scottish Oceans Institute, Gatty Marine Laboratory, School of Biology, University of St Andrews, Fife, KY16 8LB Scotland, UK; 10 Scottish Association for Marine Science, Oban, Argyll and Bute, PA37 1QA Scotland, UK; 11 University of Washington, Seattle, WA 98195 USA; 12 Department of Applied Marine Ecology and Modeling, Aarhus University, Nordre Ringgade 1, 8000 Aarhus C, Denmark; 13 German Federal Institute of Hydrology, Department for Microbilogy, Am Mainzer Tor 1, 56068 Koblenz, Germany

**Keywords:** Arctic ecosystem, Arctic Ocean, Light transmission, Phytoplankton, Primary production, Sea ice algae

## Abstract

The Arctic marine ecosystem is shaped by the seasonality of the solar cycle, spanning from 24-h light at the sea surface in summer to 24-h darkness in winter. The amount of light available for under-ice ecosystems is the result of different physical and biological processes that affect its path through atmosphere, snow, sea ice and water. In this article, we review the present state of knowledge of the abiotic (clouds, sea ice, snow, suspended matter) and biotic (sea ice algae and phytoplankton) controls on the underwater light field. We focus on how the available light affects the seasonal cycle of primary production (sympagic and pelagic) and discuss the sensitivity of ecosystems to changes in the light field based on model simulations. Lastly, we discuss predicted future changes in under-ice light as a consequence of climate change and their potential ecological implications, with the aim of providing a guide for future research.

## Introduction

The reduction of Arctic sea ice is one of the strongest manifestations of global climate change. Besides shrinking in extent, sea ice properties are also changing, as the Arctic Ocean shifts towards a thinner (e.g. Renner et al. 2014) and younger (Maslanik et al. 2011) ice pack. This shift has profound implications for the structure of the remaining sea ice, for melt pond development, and for the amount of snow that accumulates. The open water season has become longer because sea ice is forming later and melting earlier (e.g. Stroeve and Notz 2018). These changes in sea ice strongly modulate the underwater light field (i.e. its intensity and spectral composition) leading to an increase in light penetrating through the ice cover into the water (Nicolaus et al. 2012).

Light and nutrients are key drivers of Arctic ecosystem dynamics. Primary producers within the sea ice (sea ice algae) and in the underlying ocean (phytoplankton) require light for growth (e.g. Michel et al. 1988). Therefore, changes in light availability can have a significant impact on Arctic primary production. Ice algae and phytoplankton form the basis of the Arctic marine food web, thus changes in primary production will have cascading effects on higher trophic level species such as fish, birds, and mammals (e.g. Steiner et al. 2019). However, food web responses may not be linear since the timing, as well as the magnitude of production are important. Mismatches in the timing of the blooms of sea ice algae and phytoplankton—principally regulated by light—and the timing of the zooplankton bloom (termed secondary production)—principally regulated by temperature (Richardson 2008)—may decouple primary and secondary production, with consequences for fish and higher trophic levels (Søreide et al. 2010). Furthermore, many higher trophic level predator–prey interactions are themselves regulated by light (e.g. Hobbs et al. 2021), as the ability of visual predators (e.g. fish, birds) to detect prey is a function of available light (as well as visual acuity and prey size).

**Fig. 1:**
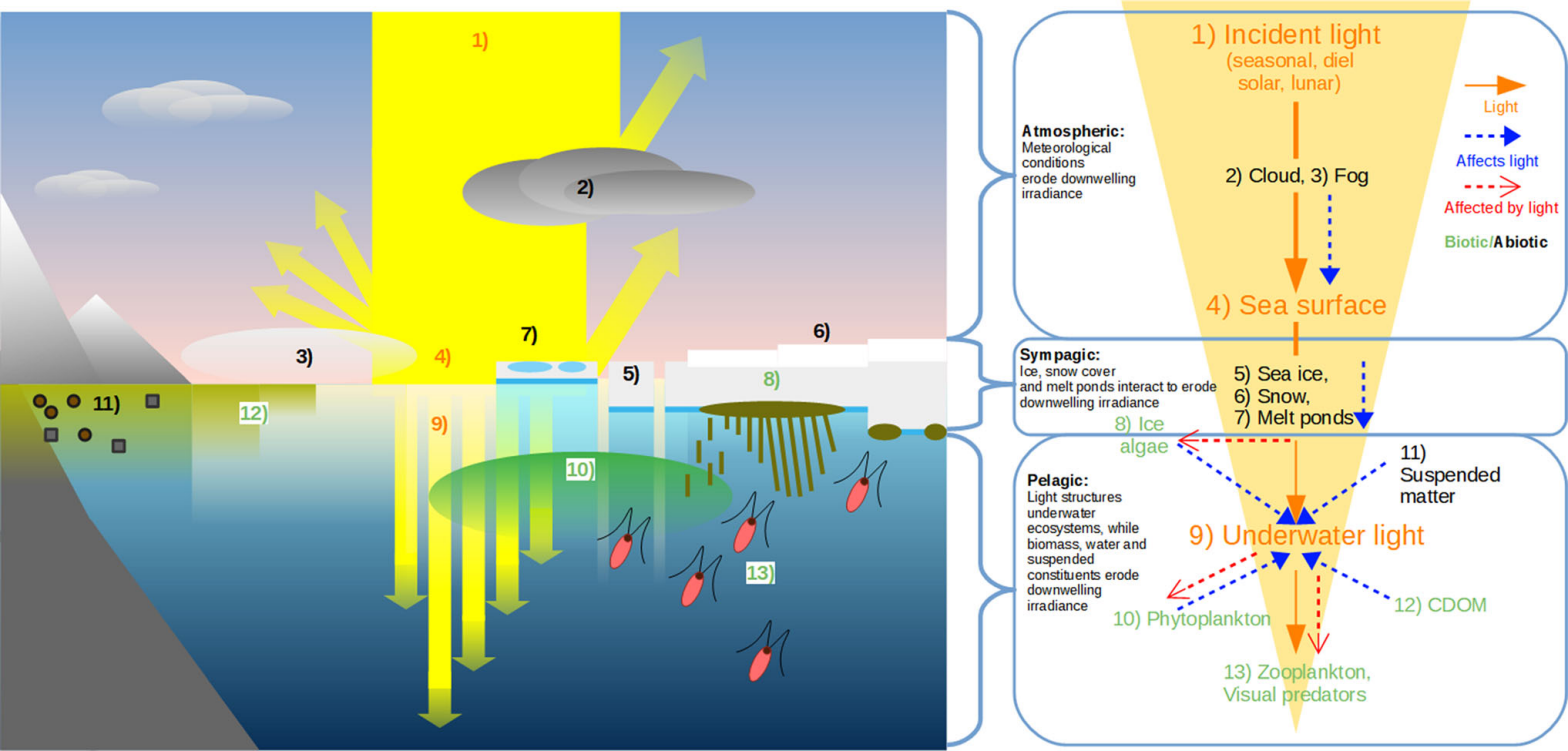
Schematic of light transmission through atmosphere, sea ice and snow, and ocean (credit: Andrew Orkney)

Monitoring under-ice light levels and ecosystem responses is crucial for better understanding the effects of ongoing changes on sympagic (ice associated) and pelagic (water column) Arctic ecosystems. Obtaining observations in extreme conditions, which are characteristic of the polar environment, remains a challenge. We are, therefore, largely reliant upon numerical models and satellite products to quantify large-scale changes in the light field and to predict associated ecological implications. Recent modelling studies have shown a marked increase in light conditions favourable for under-ice blooms over the last two decades (Horvat et al. 2017) and pointed to the controlling role that shortwave radiation has on the magnitude of phytoplankton bloom (Popova et al. 2010). Moreover, CMIP5 simulations (Tedesco et al. 2019) indicate sympagic primary production, which is triggered by light availability, will begin earlier and increase at most latitudes under modelled climate change scenarios. Thus, the parameterization of the under-ice light field in numerical models is crucial to properly represent future trends in high-latitude ocean primary production. In this study, we review the physical and biological processes that alter and attenuate light in its journey through the atmosphere, the snow and ice cover, and the upper ocean (“Incoming light” to “Light propagation through seawater—the role and sources of CDOM and SPM” sections, Fig. 1). Focus is on the parameterizations of light transmission through sea ice and snow which are often used in large-scale sea ice-ocean models (e.g. CMIP6, Eyring et al. 2016) and recently applied to satellite data (Stroeve et al. 2021). We elucidate the role of primary producers on light absorption, as well as their dependency on light (“Sea ice algae and phytoplankton” section). By means of numerical simulations (“Sensitivity of sea ice algae and phytoplankton to light transmission parameterization” section), we show how the choice of light transmission parameterizations and their parameters affect the simulated under-ice light field and, as consequence, the sympagic and pelagic ecosystems. Finally, we consider possible societal implications of future changes in the Arctic (“Expected future changes and socio-economic impact” section).

## Incoming light

**Fig. 2:**
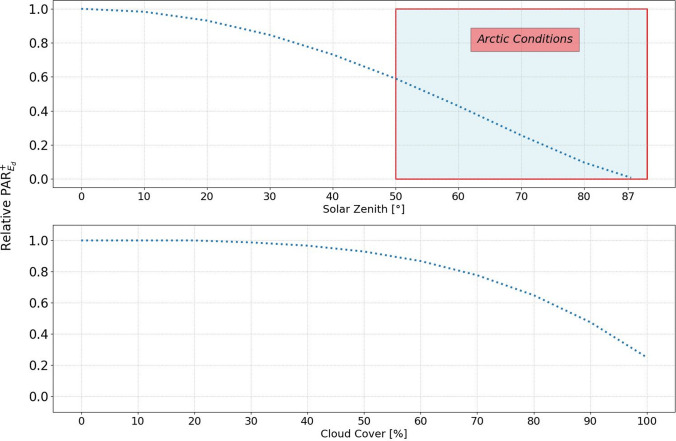
Effect of varying solar zenith angle (upper panel), and cloud cover (lower panel) on surface PAR irradiance. Data are presented as the ratio of light intensity relative to the equator at noon on midsummer’s day. The red box highlights solar zenith angles relevant for Arctic regions. All simulations done with Hydrolight Radiative Transfer Software that includes the RADTRAN sky model (Gregg and Carder 1990) and the cloud model from Kasten and Czeplak (1980)

Light availability at the ocean surface in the Arctic is primarily governed by seasonal changes in solar zenith angle and cloud cover (Fig. 1). In the range relevant for polar applications, incoming light decreases almost linearly with solar zenith angle above 50 degrees as illustrated in Fig. 2, upper panel. Clouds can be a dominant feature in the Arctic. Their impact remains limited for cloud cover up to ~ 30%, but it rapidly reduces surface irradiance for higher levels of cloud cover (Fig. 2, lower panel). 100% cloud cover reduces surface irradiance to ~ 20% of cloud free levels.

In coupled physical ecosystem models (e.g. SINMOD, Wassmann et al. 2006; BESTMAS + CLADACH, Banas et al. 2016) incoming light is often provided as shortwave radiation (300–3000 nm) by satellite available products and reanalysis data that usually include atmospheric conditions such as cloudiness. The part of the spectrum relevant for biological processes is termed PAR (Photosynthetically Active Radiation: 400 nm–700 nm) which is generally taken in the range 43–50% of the provided shortwave radiation (e.g. Banas et al. 2016; Castellani et al. 2017; Schourup-Kristensen et al. 2018).

## Light attenuation by sea ice

Despite the notably high albedo (i.e. fraction of solar radiation reflected by the surface) of a sea ice cover, a fraction of the light is propagated through the ice, into the ocean. Sea ice attenuates light about ten times more strongly than clear ocean waters and in turn, snow attenuates ten times more strongly than the ice (Perovich 1996). Consequently, the under-ice light field averaged over large scale is only up to 20% of the incoming light (Katlein et al. 2019). Besides the thickness of the snow and ice cover, light transmission is strongly influenced by the internal structure of the ice and snow, and by the different surface elements that characterise the ice cover in different seasons (e.g. pressure ridges, melt ponds). While the large ice thickness in ridges reduces light levels underneath, their complex internal structure can lead to favourable light conditions within (Katlein et al. 2021). Melt ponds on top of the ice can dramatically increase the transmittance of sea ice since they have a much lower albedo than the surrounding ice (Nicolaus et al. 2012; Light et al. 2015). Melt ponds can also introduce strong horizontal inhomogeneity to the light field in the water column (Frey et al. 2011). Further constituents deposited on top of the ice surface or within the ice matrix like sediment, Colored Dissolved Organic Matter (CDOM), soot, or algae can locally or temporally influence the intensity and spectral shape of the transmitted light.

### Modelling light transmission through sea ice

In numerical ice-ocean models, such as those contributing to CMIP6 (Eyring et al. 2016), light attenuation in sea ice is represented at different levels of complexity. In the presence of sea ice, the amount of underwater light in each grid cell is the weighted average of light reaching the ice-free ocean surface and light transmitted through the ice cover. The simplest approach assumes zero transmittance for ice (bare, ponded, or snow-covered) and 0.93 (the difference between 1—maximum albedo—and an albedo of 0.07, the value which is typically used for open water) transmittance for areas of open water (Perovich et al. 2007). More complex models use a variety of approximations and numerical techniques for computing light transmission in the ice-ocean domain. One such approach widely used in CMIP6 models, relies on a simple exponential description (Grenfell and Maykut 1977) with empirically derived extinction coefficients that represent the attenuation of light through snow and ice. The values of extinction coefficients vary according to season and surface characteristics. The value for bare ice is usually taken in the range 1.1 to 1.6 m^−1^ (Perovich 1996; Grenfell and Maykut 1977), whereas the values for snow show a larger variability with values ranging from 4.3 to 40 m^−1^ (Perovich 1996). In the case of bare summer ice, the absorption of solar radiation causes the above-freeboard ice to weather, become crumbly, and have a significantly larger air-ice interface, thus scattering light much more effectively than the ice beneath. The uppermost portion of this layer is termed the “surface scattering layer” (SSL), which is typically up to 10 cm thick (e.g. Light et al. 2008). To estimate light transmittance through the sea ice cover, the simple exponential models rely on an approximation that extinguishes a significant portion (up to 70%) of the incident light in the SSL. Thin ice with small freeboard, and in turn a rather thin SSL, forms a relevant fraction of the ice cover, especially in the marginal ice zone and in the seasons of ice formation/melt. Thus, the treatment of the SSL and of the light transmission through thin ice in models will impact the under-ice PAR, and consequently, the ecosystems (“Sensitivity of sea ice algae and phytoplankton to light transmission parameterization” section).

More sophisticated approaches include explicit treatment of multiple scattering and use inherent optical properties to compute the full radiation budget for all surface types present (Briegleb and Light 2007). Indeed, snow-covered ice, ponded ice, melting ice, and even bare ice all exhibit large vertical gradients in scattering. Such treatments are incorporated into only a few sea ice models (e.g. CICE, Holland et al. 2012).

## Light propagation through seawater—the role and sources of CDOM and SPM

Once light reaches the ocean surface, it is further attenuated through absorption and scattering by sea water itself and by particles such as CDOM, Suspended Particulate Matter (SPM), and phytoplankton (“Sea ice algae and phytoplankton” section). Within the visible spectrum, absorption by pure water is two orders of magnitude stronger in the red than in the blue (e.g. Pope and Fry 1997), whereas scattering is an order of magnitude greater in the blue than in the red. The scattering coefficient also depends on the salinity of the water, with values increasing by around 30% from freshwater to sea water (Morel 1966). CDOM primarily affects light propagation through absorption (Dall'Olmo et al. 2009), with CDOM absorption decreasing approximately exponentially from the blue to the red (e.g. Carder et al. 1989). Similarly, SPM absorbs highly in the blue and decays almost exponentially towards the red (Babin et al. 2003). Scattering by SPM exhibits similar behaviour in both organic and mineral form, with limited spectral variability in scattering coefficients (Lo Prejato et al. 2020).

CDOM in the Arctic is mainly provided by discharge from rivers. In open water, sources of SPM are mainly organic detritus, formed during phytoplankton blooms (Macquaker et al. 2010). In coastal regions, the sources of SPM are mainly mineral in origin, usually originating from coastal erosion, or run-off from rivers and land (Klein et al. 2019).

On a global scale, CDOM and SPM absorption in the blue is broadly equivalent to that of phytoplankton (Siegel et al. 2002). However, light attenuation by these constituents in marine ecosystem models is generally represented by very simplistic parameterizations. Commonly, attenuation in water in Arctic ecosystem models is a function of phytoplankton biomass with a constant background PAR attenuation, while the effect of CDOM/SPM in some instances is added as an average over a broad, heterogeneous ocean region (e.g. Banas et al. 2016). Other models represent attenuation by SPM and other dissolved organics as a function of salinity (e.g. Mei et al. 2010). However, in coastal regions, such as the Russian shelves highly affected by river discharge, the lack of an explicit treatment of CDOM might lead to biases in model simulations (e.g. Schourup-Kristensen et al. 2018).

## Sea ice algae and phytoplankton

Light transmitted within or under sea ice may be absorbed or scattered by phytoplankton and ice algae (Kirk 1994). The absorption of visible radiation by algal cells is spectrally dependent and relies on the presence of a range of chlorophyll and carotenoid molecules and biliproteins, each with their own characteristic absorption spectrum. There are also strong differences in the efficiency of light absorption by pelagic and sympagic algae based on their intracellular packaging of pigment molecules and the size and morphology of the cells (e.g. Chase et al. 2013). It is the harvesting of sunlight by algal pigments that ultimately powers the metabolism of primary producers. In contrast to absorption, resulting mainly from chemicals stored in the cell interior, the amount, wavelength dependence, and direction of scattering by phytoplankton cells depends on their size and exterior morphology.

Whilst there exists variation in the bio-optical properties of algal cells, the most important component of the light absorption spectra is the photosynthetic pigment chlorophyll *a* (chl *a*). Thus, the light absorptive properties of ice algae and phytoplankton, and the effect they have on the transmission of light through the water column, can largely be described as a function of chl *a* concentration. However, this may not be true for coastal waters, where CDOM derived from the land may enter the sea (“Light propagation through seawater—the role and sources of CDOM and SPM” section).

### Photophysiology of Arctic algal assemblages—theoretical and modelling approach

**Fig. 3:**
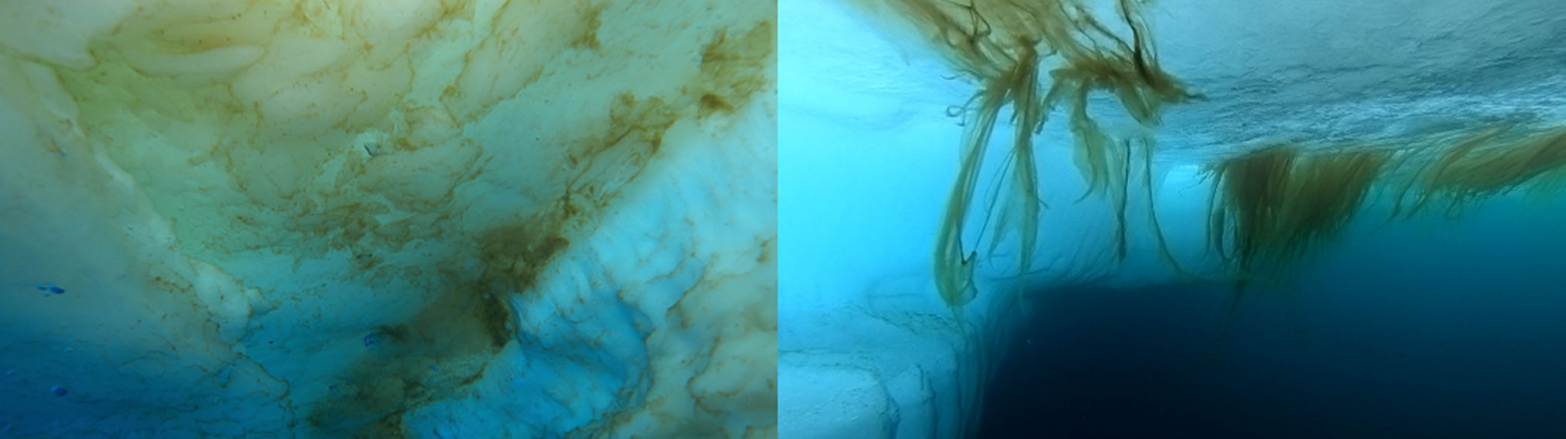
Example of sea ice algae forming a mat-like colony under the ice (left panel, credit: Carsten Wancke), and of *Melosira arctica* forming filaments affixed to the sea ice subsurface (right panel, credit: Oliver Müller)

The ways in which different algal assemblages interact with incident light fields vary. Hence, spatiotemporal variability in both the intensity and the spectral quality of light may favour some communities over others. Many Arctic algal communities also adopt different photophysiological strategies to cope with the highly variable light field in the Arctic. Despite the relief of darkness in spring, ice algae and many phytoplankton communities find themselves shaded beneath ice and snow. Shade-adapted algae include the diatoms *Nitzschia frigida*, the main species dominating sea ice algae communities, and *Melosira arctica* (Fig. 3). Such sympagic diatoms form mat-like colonies or filaments affixed to the sea ice subsurface. Despite very low light intensity, high pigmentation and occupying a stable band a few centimetres thick at the ice-water interface permit growth as early as February (Syvertsen 1991). During the summer months, many Arctic ice algae and phytoplankton assemblages have unique adaptations that let them survive relatively high light in summer without sacrificing their ability to grow in lower light in spring. An example are the photophysiological adaptation strategies of diatom species: their intense pigmentation and concentrated communities result in self-shading, moderating their light environment (Barros et al. 2003). The ability of diatoms to adapt to a range of light environments, ranging from light-limited conditions beneath seasonal ice to intensely illuminated melt layers explains their widespread occurrence in both under-ice and ice-edge blooms (Degerlund and Eilertsen 2010).

Once a photon is absorbed by an algal cell, how efficiently the photosynthetic machinery of the cell can convert this into chemical energy (photophysiology) depends on several factors. Variation in algal photophysiology has been explicitly considered in Arctic ecosystem models for at least three decades (Slagstad and StØle-Hansen 1991). Many recent high-latitude models have represented the phytoplankton by two competing size classes (usually taken to represent diatoms and small flagellates), either with (Wassmann et al. 2006) or without (Vernet et al. 2017) including differences in photophysiology between the two groups. Banas et al. (2016) found that including seasonal photoacclimation in a one-phytoplankton model was sufficient, and also necessary, to capture the timing and magnitude of a high-latitude spring bloom in detail. The above cited model studies illustrate that the inclusion of flexible photophysiology within populations as well as photoresponse-based competition between functional types is required in high-latitude plankton models to properly represent timing and magnitude of the bloom.

## Sensitivity of sea ice algae and phytoplankton to light transmission parameterization

Modelled PAR and growth of sea ice algae and phytoplankton are sensitive to light transmission parameterizations. In the case of the exponential model, largely used in CMIP6 models, the same holds for the chosen extinction coefficients which determine the exponential decay in sea ice and snow, both of which are derived from observations, but are subject to large uncertainty (Katlein et al. 2019; Castellani et al. 2020). To illustrate the consequence of different treatments of light transmission through thin ice and through the SSL, and of the extinction coefficients, we present results of numerical experiments performed with the Finite Element Sea ice Ocean Model (FESOM) version 1.4 coupled to the ocean biogeochemical model REcoM2 (Schourup-Kristensen et al. 2018) and to the Sea Ice Model for Bottom Algae SIMBA (Castellani et al. 2017).

Experiment ‘standard’ assumes the standard thickness of the SSL of 10 cm (Light et al. 2008) for sea ice. Experiment ‘drainage’ investigates the impact of making the existence and thickness of the SSL dependent on the freeboard, assuming a linear increase of the SSL from 0 (for ice thickness below 50 cm) up to 10 cm (for sea ice thickness of 80 cm). The third experiment ‘drainage_2k_s_’ investigates the effect of a doubled extinction coefficient for dry snow k_s_ from 10 to 20 m^−1^. To highlight the sensitivity of the biological system to changes in light transmission parameterization, we focus on the seasonal evolution of in-ice chl *a* (as proxy for sea ice algae), and diatom net primary production (NPP) in the ocean (phytoplankton).

**Fig. 4:**
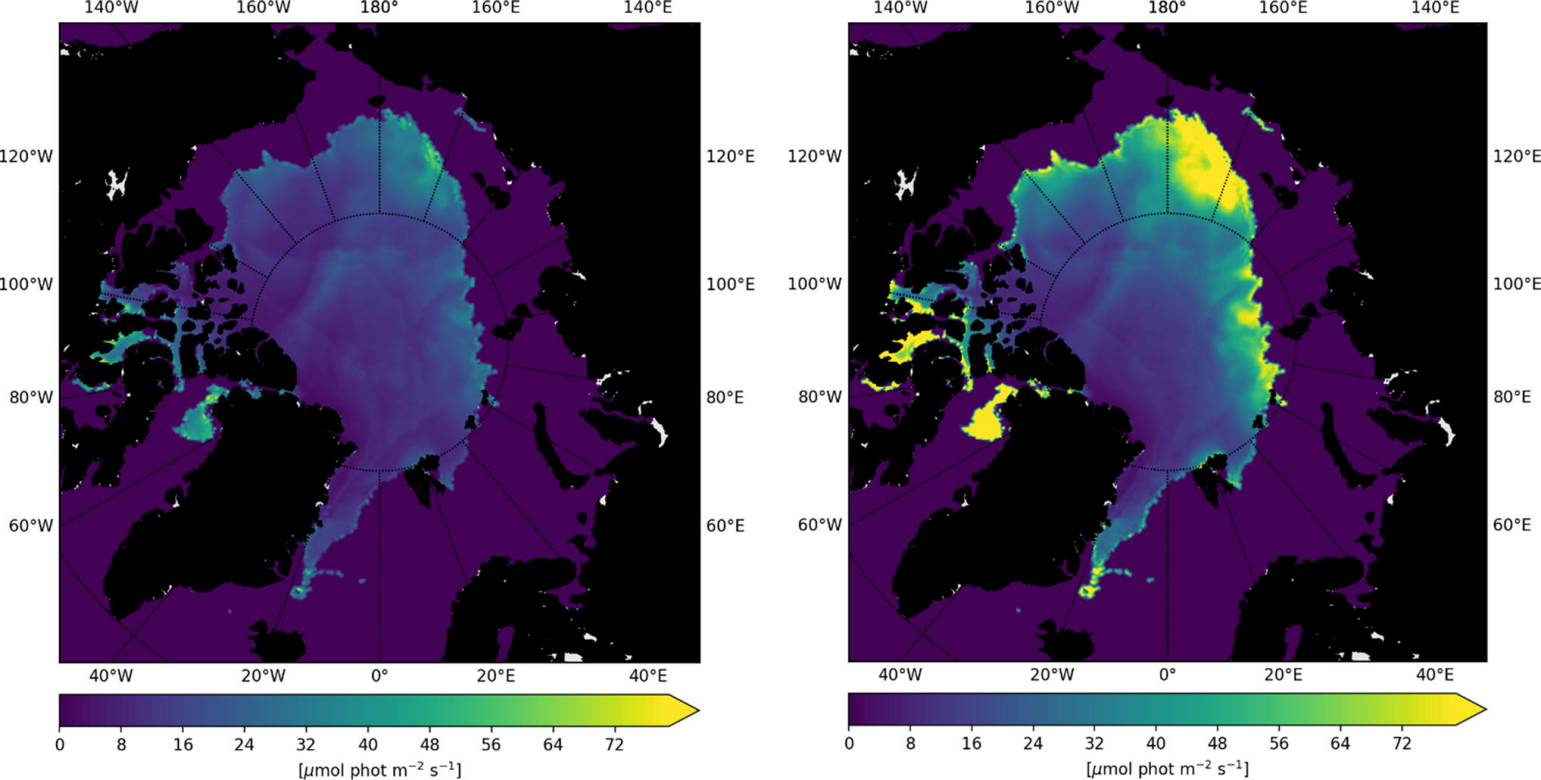
Under-ice PAR at the end of August 2012 for the case with a standard parameterization of the SSL (‘standard’ simulation, left hand side) and for ‘drainage’, when the SSL only exists if the sea ice is thicker than 50 cm (right hand side)

**Fig. 5:**
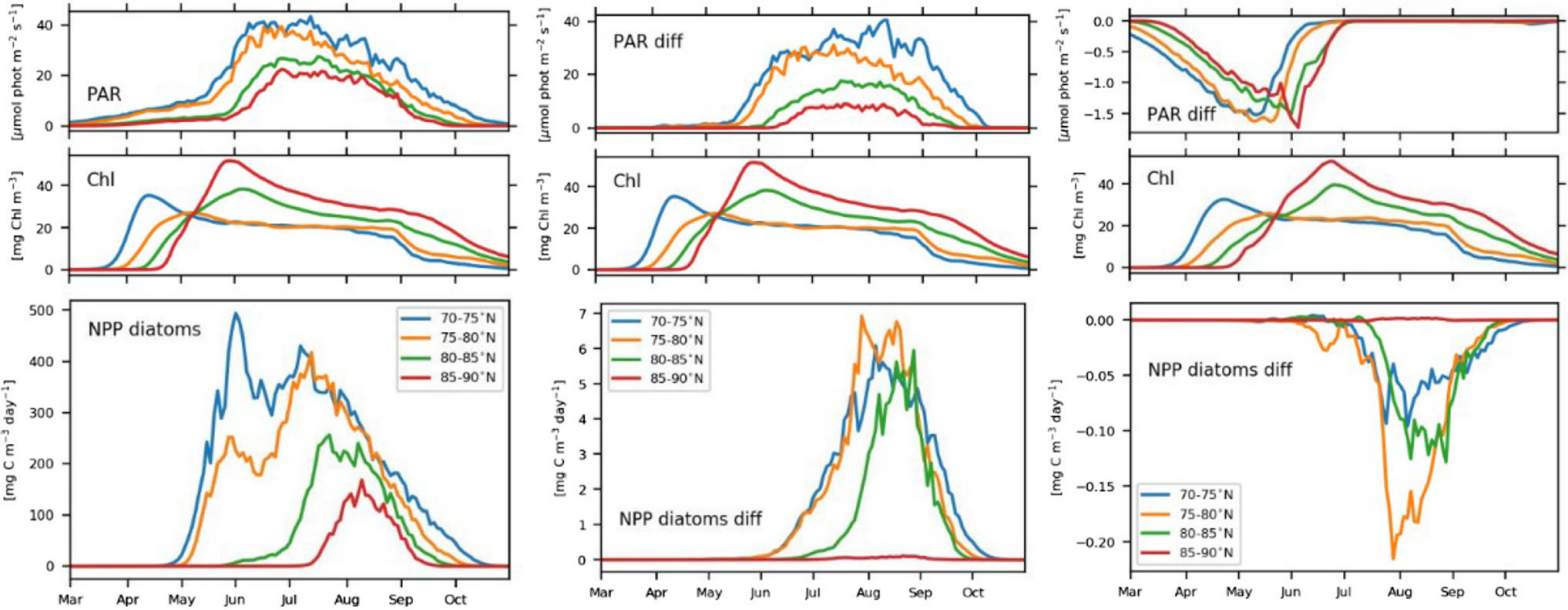
Mean PAR (top row), mean sea ice algae chl *a* (middle row) and NPP of diatoms (bottom row) in latitude bands. The columns show on the left hand side: the standard parameterization of the SSL (‘standard’ simulation); in the middle: ‘drainage’—existence of an SSL only if the sea ice thickness > 50 cm; and on the right hand side: ‘drainage_2K_s_’ with a doubled extinction coefficients for snow K_s_. For the simulations ‘drainage’ and ‘drainage_2k_s_’, NPP and PAR are shown as differences ‘drainage’—‘standard’ and ‘drainage_k_s_’—‘drainage’, respectively

The introduction of a thickness-dependent SSL (‘drainage’) leads to a large increase of PAR in the summer months (Figs. 4 and 5). Particularly in the marginal areas, where ice is often thinner than 80 cm, under-ice PAR increases up to twice that in the ‘standard’ simulation (Fig. 4). Differences in PAR start to appear in May–June (Fig. 5) and lead to a small increase in diatom NPP, limited to lower latitudes (70–85°N), but with no effect on the onset of the bloom. Differences remain negligible for sea ice algae. In contrast, a doubling of the extinction coefficient for snow (‘drainage_2xKs’) already affects PAR in spring and early summer (Fig. 5), leading to a reduction of under-ice PAR in those months when snow did not yet melt completely. Using a larger k_s_ causes a delay of the sea ice algal bloom onset by up to 2 weeks in higher latitudes and a shift of the peak by more than a month (Fig. 5). The effects on the phytoplankton remain, however, negligible, since phytoplankton start to grow later in the season compared to sea ice algae, when the differences in PAR become negligible.

Our results show that the choice of parameterizations of light transmission and their coefficients affect underwater light, but differently according to season. The response of the ecosystems also differs, with sea ice algae being more sensitive than phytoplankton. Moreover, sea ice algae and phytoplankton have different photophysiology (“Sea ice algae and phytoplankton” section), which results in different timing of the bloom and thus different response to changes in the light field. These results show that how reliably we can project future changes in the magnitude and particularly in the timing of sea ice and ocean primary production is affected also by how we describe light transmission in models. With the aim to reliably project future changes in sympagic and pelagic productivity, there is the need to theoretically constrain parameterizations (such as the representation of the SSL) and their parameters. Moreover, an intercomparison between model results adopting different parameterizations and parameters is needed in order to quantify the differences between formulations and their impact on ecosystems response.

## Expected future changes and socio-economic impact

The current global warming trend is likely to result in future increase of light reaching the upper ocean (Lannuzel et al. 2020) with consequences for the temporal and spatial patterns of sea ice algae and phytoplankton growth. Phytoplankton growth is expected to shift northwards and occur earlier following a reduction in sea ice extent and thickness (Ardyna and Arrigo 2020). Climate models suggest that Arctic precipitation will transition from being snow to rain dominated leading to a reduced snow cover (Bintanja and Olivier 2017) and the increased likelihood that the sea ice algal bloom will happen earlier in the season (Post et al. 2013; Tedesco et al. 2019; Lannuzel et al. 2020). These shifts have the potential to significantly alter the composition and abundance of primary producers by favouring different photoadaptation strategies. Furthermore, such potential shifts in timing are likely to cause a mismatch between primary production and associated zooplankton grazing, thus compromising the life cycle of zooplankton (Søreide et al. 2010).

Changes in the light field may also modify the vertical positioning of zooplankton as they negotiate the trade-offs between predation risk and feeding (Hobbs et al. 2021). Such migratory behaviour of zooplankton can actively draw carbon out of the surface waters making it an important contribution to the vertical carbon flux (Hansen and Visser 2016). Thus, a consequence of a change in light-mediated vertical migration is disruption of the biological pump.

The most substantial impacts on fish and seabirds are likely to be through visual predation. Increasing illumination will improve feeding conditions for epipelagic (i.e. that oceanic zone where enough light penetrates for photosynthesis) fish, and increased predation by fish may lead to changes in the size distribution of the zooplankton community (Varpe et al. 2015). Planktivorous fish themselves will be subject to increased predation risk, so increasing illumination may lead to wholesale changes in trophic transfer efficiency through food webs (Langbehn and Varpe 2017). However, observations and models both suggest that in some high-latitude regions the increases in primary production associated with low-ice conditions are actually inversely correlated with the success of fish, birds, and mammals, for reasons of plankton composition and timing (Banas et al. 2016). This gives the impression of a fragile ecosystem, but there are internal balances that may come into play to provide resilience in the form of variable life history strategies (Hobbs et al. 2020) and changes in species composition (Renaud et al. 2018) such that Arctic marine food webs may be more resilient to climate-related shifts than previously assumed.

Changes in both the physical environment (e.g. sea ice loss in coastal regions, loss of permafrost) and the ecosystem will affect ecosystem services, with strong societal and economic effects (O’Garra 2017). This is particularly relevant for communities based on fisheries, subsistence hunting and coastal infrastructure, but also on commercial activities making use of those ecosystem services. Further warming and ice loss may lead to further biogeographic shifts in fish distributions and perhaps to behavioural changes such as reduced school sizes (Brierley and Cox 2010). Following fish, larger Arctic species such as seals and whales are moving northwards into the Arctic basin. This will affect native populations, who rely on whales as a food source, as well as for their cultural heritage.

Increasing light intensity might lead via altered primary production to altered fish production, including a predicted increase in pelagic and planktivorous fish (Heath et al. this volume). There is growing concern that the next big global development in commercial fishing will target the mesopelagic, where the estimated c. 10 GT fish biomass (Irigoien et al. 2014) may be the planet’s last remaining untapped source of protein. Fishing in the central Arctic Ocean is, however, prohibited until at least 2034 by binding legal agreement between multiple Arctic nations (Hoag 2017).

The complexity and interconnectivity of the social-ecological system in the Arctic, at a time where it experiences rapid changes, is thus calling for holistic studies to assess the impacts on the ecosystems and on human communities, as well as ways to respond.
